# Comparative morphology of the spermatheca in Megalopodidae (Coleoptera, Chrysomeloidea)

**DOI:** 10.3897/zookeys.720.14088

**Published:** 2017-12-11

**Authors:** Geovanni M. Rodríguez-Mirón, Santiago Zaragoza-Caballero, Sara López-Pérez

**Affiliations:** 1 Departamento de Zoología, Instituto de Biología, Universidad Nacional Autónoma de México, A.P. 70-153, 04510 Mexico City, Mexico

**Keywords:** Female genitalia, Zeugophorinae, Megalopodinae, Palophaginae, flagellum, taxonomic significance

## Abstract

The spermatheca is an organ that stores and maintains viability of sperm until fertilization. It has an important role in copulation and oviposition, and it is highly informative in species delimitation. Here, we present a comparative study of the spermathecal morphology in the coleopteran family Megalopodidae. The spermathecae of 34 species, representing 13 genera and all three subfamilies, were studied. Illustrations are newly provided for all species, except in 14 cases in which illustrations were reproduced from previously published literature. Our results show that each subfamily of Megalopodidae can be effectively differentiated based on the particular spermathecal anatomy. In addition, the spermathecal anatomy presents a range of variation within each subfamily, useful for diagnosing species and, in some cases, identifying groups of genera. For instance, the “American group” is thus recognized in this study.

## Introduction

The female internal reproductive organs in insects consist of several organs: a pair of ovaries with their respective oviducts, a median ectodermal tube, a vagina, a bursa copulatrix and the spermatheca (Snodgrass 1935, Suzuki 1988, Triplehorn et al. 2005). The spermatheca (multiple spermathecae in some instances) is an invagination of the eighth abdominal segment (Snodgrass 1935); and its shape and number depend on the group of insects (Harterreiten-Souza and Pujol-Luz 2012, Pascini and Martins 2017). The spermatheca is an important organ that stores and maintains viability of sperm until fertilization, and it has an important role in copulation and oviposition (e.g. Gschwentner and Tadler 2000, De Marzo 2008, Harterreiten-Souza and Pujol-Luz 2012, Pascini and Martins 2017).

**Figure 1.**
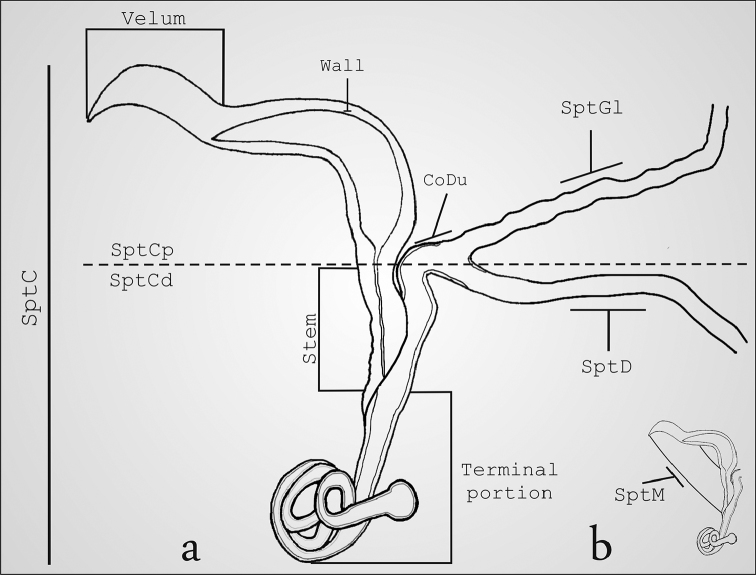
Structure of the spermatheca in Megalopodidae (*Mastostethus novemaculatus*). **a** general view, **b** spermathecal muscle.

The order Coleoptera exhibits five patterns of spermathecal morphology (De Marzo 2008). These patterns are distinguished by the presence, absence or variations of the following structures: spermathecal capsule, spermathecal duct, and spermathecal gland (De Marzo 2008). The most widespread pattern is to have only one spermathecal capsule that stores sperm, and this capsule is connected with the bursa copulatrix by one spermathecal duct that allows the sperm to be transported to the spermathecal capsule after copulation (Gack and Peschke 1994, De Marzo 2008). In addition, there is only one spermathecal gland that secretes glycoproteins responsible for the migration of sperm from the bursa copulatrix to the spermathecal capsule (Fig. 1a) (Aslam 1961, Grodner and Steffens 1978, Suzuki 1988, De Marzo 2008, Matsumura and Suzuki 2008). Finally, the distal and proximal portions of the spermatheca are connected by a muscle (Fig. 1b), the contraction of which causes the sperm to be transferred to the bursa copulatrix (Rodríguez 1994).

Classification systems have mainly utilized characters of the external morphology, such as wing venation; however, most of these classifications change constantly because of symplesiomorphy and homoplasy within these character sets. Therefore, taxonomists have studied internal morphology and genital features, which, in combination with the features mentioned above, will contribute to a more stable classification (Aslam 1961, Kasap and Crowson 1979, Mann and Crowson 1983, Suzuki 1988, Wanat 2007, Santos and Rosado-Neto 2010). Male genitalia have been widely used to differentiate species, even between closely related taxa, because of their rapid divergence due to sexual selection (Arnqvist 1997, Flowers and Eberhard 2006, Zunino 2012).

The female internal reproductive organs have been used less frequently; however, they have also been found useful in diagnosing certain groups (Hernández and Ortuño 1992, Hernández 1993, Ferronato 2000, Gaiger and Vanin 2008). Histological differences and shape variations are useful in distinguishing species, species groups and even genera (Suzuki 1988, Candan et al. 2010). These variations render the spermatheca as a character complex with high taxonomic value. For example, spermathecal features have been used to separate and diagnose the tribes of Scarabaeinae (López-Guerrero and Halffter 2000); in Curculionidae and Carabidae, the spermathecae also allow the recognition of species and genera (Aslam 1961, Schuler 1963).

The spermatheca in Chrysomeloidea has been useful to define subfamilies, genera, species, and groups of species (Reid 1989, Hernández 1993, Biondi 2001, Borowiec and Świętojańska 2001, Borowiec and Skuza 2004, Borowiec and Opalinska 2007, Yus-Ramos 2008, Borowiec and Pomorska 2009, Bi and Lin 2013, López-Pérez et al. 2016, Rodríguez-Mirón and Zaragoza-Caballero 2017). Suzuki (1988) presented the first comprehensive study of the male and female genitalia of Chrysomelidae, and he described the spermathecae of two species of Megalopodidae, *Zeugophora annulata* (Baly, 1873), and *Temnaspis japonica* Baly, 1873. This author included these genitalic features in a phylogenetic analysis, proposing Megalopodinae and Zeugophorinae as sister taxa, and placing both subfamilies within Chrysomelidae. In a later study, Megalopodidae was ranked as a separate family based on larval anatomy, and the spermathecae of some species of Palophaginae were illustrated and described (Kuschel and May 1990, 1996).

Other megalopodid taxa that have had their spermathecae described and illustrated are: *Mastostethus* Lacordaire, 1845, *Agathomerus* Lacordaire, 1845, and *Megalopus* Fabricius, 1801 (Suzuki 2003). Additionally, Reid (1989, 1992, 1998) illustrated the spermathecae of *Zeugophora vitinea* (Oke, 1932), *Zeugophora williamsi* Reid, 1989, *Zeugophora javana* Reid, 1992, and *Zeugophora toroja* Reid, 1998. Finally, Sekerka and Vives (2013) described and illustrated the spermatheca of *Zeugophorella riedeli* (Medvedev 1996).

Megalopodidae currently consists of 552 described species, which are classified into three subfamilies (Megalopodinae, Zeugophorinae, and Palophaginae) (Rodríguez-Mirón 2016). However, the spermathecae of only 5% of these species have been described. Herein, we describe and compare 34 species, representing 13 genera and two subgenera for one of these genera. This work presents a panorama of the diversity and complexity of the spermathecal capsule in Megalopodidae, with the objective of shedding light in future taxonomic and phylogenetic studies.

## Methods

The spermathecae of 34 species of Megalopodidae were examined. These species represent three subfamilies, 13 genera and two subgenera for one genus. Approximately 100 specimens were examined, distributed between the 34 species studied (Table 1). Illustrations from Suzuki (1988, 2003), Kuschel and May (1990, 1996), Reid (1989, 1992, 1998) and Sekerka and Vives (2013) were reproduced in the present study and were used to establish putative homologies among these structures.

**Table 1.** Species studied.

Species	Geographic information in label	No. specimens
**Megalopodinae**		
Agathomerus (Agathomeroides) flavomaculatus (Klug, 1824)	Brazil	4
Agathomerus (Eugathomerus) sellatus (Germar, 1823)	Brazil	6
*Agathomerus rufus* (Klug, 1834)	Mexico	30
*Agathomerus signatus* (Klug, 1824)	Brazil	3
*Agathomerus* sp. *1	Panama	–
*Homalopterus tristis* Perty, 1832	Brazil	2
*Mastostethus hieroglyphicus* (Klug, 1834)	Mexico	9
*Mastostethus nigrocinctus* (Chevrolat, 1834)	Honduras, Costa Rica,Mexico	25
*Mastostethus novemaculatus* (Klug, 1834)	Mexico, Costa rica	6
*Mastostethus variegatus* (Klug, 1824)	Brazil	1
*Megalopus inscriptus* Klug, 1824	Peru	3
*Megalopus* sp. 1	Costa Rica	2
*Megalopus* sp. 2 *1	Panama	–
*Poecilomorpha atripes* Lacordaire, 1845	South Africa	1
*Poecilomorpha cyanipennis* (Kraatz, 1879)	South Korea, Russia	4
*Psudohomalopterus carinatus* Pic, 1920	Brazil	7
*Sphondylia* sp.	Africa	1
*Temnaspis septemmaculata* (Hope, 1831)	Laos	1
*Temnaspis japónica* Baly, 1873 *2	Japan	–
*Temnaspis* sp. *1	–	–
*Temnaspis speciosus* Baly, 1859	Bhutan, Nepal	4
**Zeugophorinae**		
*Zeugophora annulata* (Baly, 1873) *2	–	–
*Zeugophora califórnica* Crotch, 1874	USA	6
*Zeugophora indica* Jacoby, 1903	Kashmir, India	3
*Zeugophora javana* Reid, 1992 *3	Indonesia: West Java	–
*Zeugophora toroja* Reid, 1998 *4	Indonesia: West Java	–
*Zeugophora varians* Crotch, 1873	Canada, USA	4
*Zeugophora vitinea* (Oke, 1932) *5	Australia	–
*Zeugophora williamsi* Reid, 1989 *5	Australia	–
*Zeugophorella riedeli* (Medvedev, 1996) *6	New Guinea	–
**Palophaginae**		
*Cucujopsis setifer* Crowson, 1946 *7	Australia	–
*Palophagoides vargasorum* Kuschel, 1996 *8	Chile	–
*Palophagus australiensis* Kuschel, 1990 *7	Australia	–
*Palophagus bunyae* Kuschel, 1990 *7	Australia	–

*Information previously published; 1: Suzuki (2003); 2: Suzuki (1988); 3: Reid (1992); 4: Reid (1998); 5: Reid (1989); 6: Sekerka and Vives (2013); 7 Kuschel and May (1990); 8: Kuschel and May (1996).

For microscopic examination, the dried specimens were placed in hot water for 10 minutes to soften the tissues. Each abdomen was dissected along the abdominal pleura and boiled in a 10% KOH solution for five minutes. The spermatheca was dissected from the KOH preparation, washed with water, and mounted with glycerin in a glass slide for observation. Dissection and analysis were done using a Zeiss V–8 stereoscopic microscope. Photographs were made using a Zeiss Axio Zoom V–16 stereoscopic microscope equipped with an Axiocam MRC5 camera. After examination the spermatheca of each specimen was transferred to a microtube with glycerin, which was pinned underneath the specimen. The abdomen was attached to a white card using a drop of glue, also pinned underneath the specimen.

Specimens were borrowed from the following national and international museums and Institutions: BMNH–The Natural History Museum, London, U.K. (M. Geiser); MNHN–National Museum of Natural History, Smithsonian Institution, Washington, D.C., USA (A. Konstantinov); MZLU–Museum of Zoology Lund University, Lund, Sweden (Ch. Fägerström); NHMB–Naturhistorisches Museum Basel (M. Borer); CCFES–Z–Colección Coleopterológica de la Facultad de Estudios Superiores Zaragoza, UNAM, México (M. Ordóñez); CNIN–Colección Nacional de Insectos IBUNAM, UNAM, México (S. Zaragoza). Names in parentheses following each institution indicate the responsible curatorial person.

Spermathecal terminology follows Suzuki (1988) and Matsumura and Suzuki (2008) (Fig. 1). The following abbreviations are used in the descriptions and figures. SptC: spermathecal capsule; SptCp: proximal part of spermathecal capsule; SptCd: distal part of spermathecal capsule; CoDu: common duct; SptGl: spermathecal gland; SptD: spermathecal duct; SptM: spermathecal muscle.

## Results

**Figure 2.**
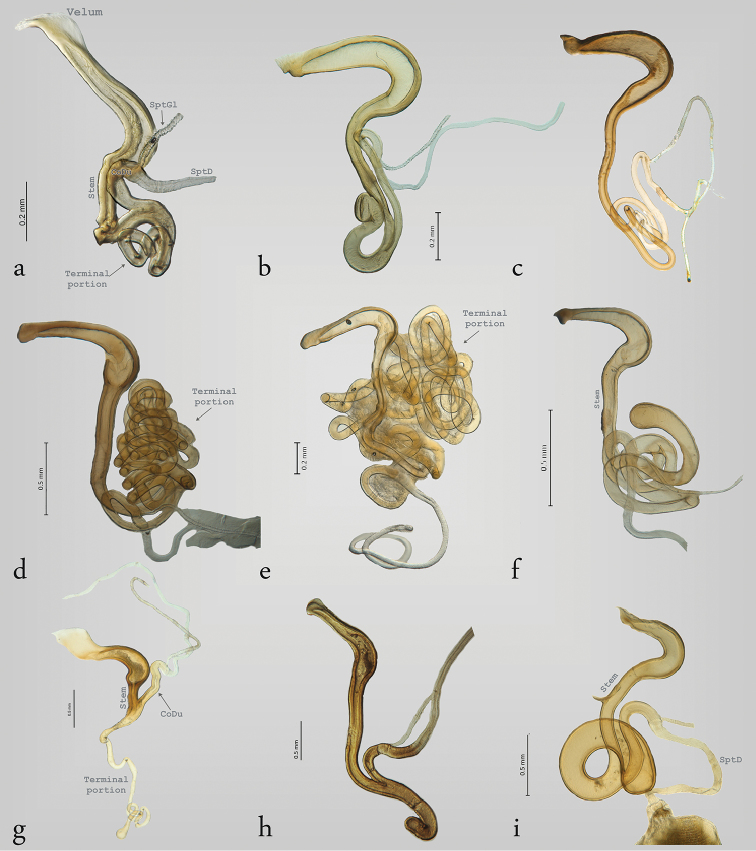
Structure of the spermatheca in Megalopodinae: *Agathomerus*, *Pseudohomalopterus*, *Homalopterus*, and *Mastostethus*. **a** *Agathomerus rufus* **b** *P. carinatus* **c** A. (Eugathomerus) sellatus **d** A. (Agathomeroides) flavomaculatus **e** *A. signatus* **f** *H. tristis* **g** *M. nigrocinctus* **h** *M. hieroglyphicus* **i** *M. variegatus*.

**Figure 3.**
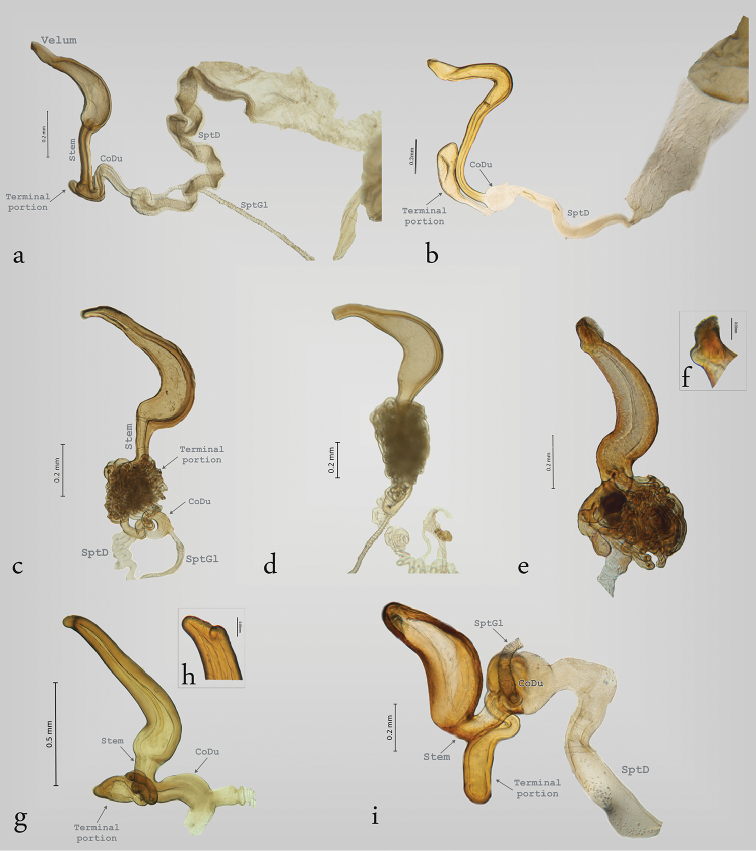
Structure of the spermatheca in Megalopodinae: *Megalopus*, *Temnaspis*, *Poecilomorpha*, *Sphondylia*. **a** *Megalopus inscriptus* **b** *Megalopus* sp. **c** *T. septemmaculata* **d** *T. speciosa* **e** *P. cyanipennis* **f** apex of the spermatheca in *P. cyanipennis* **g** *P. atripes*, **h** apex of the spermatheca in *P. atripes*, **i** *Sphondylia* sp.

**Figure 4.**
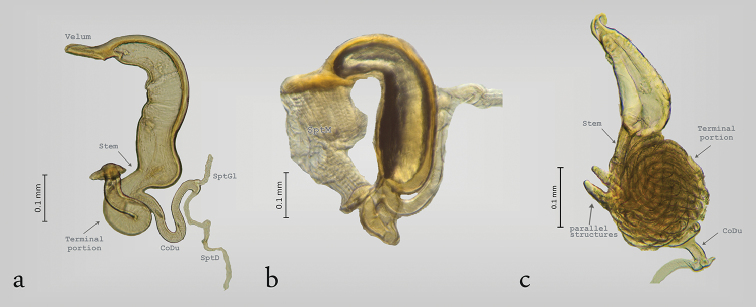
Structure of the spermatheca in Zeugophorinae **a** *Zeugophora californica* **b** *Z. varians* **c** *Z. indica*.

**Figure 5.**
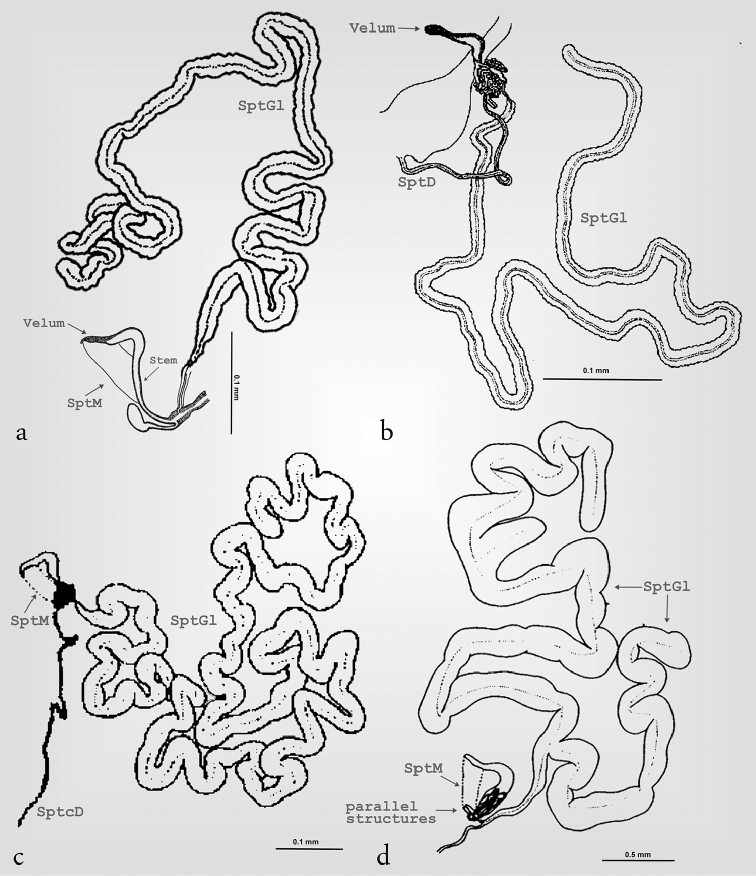
Spermatheca and spermathecal gland in Megalopodinae (**a–c**) and Zeugophorinae (**d**). **a** *Agathomerus* sp. **b** *Temnaspis* sp. **c** *T. japonica* **d** *Zeugophora annulata*. Images from Suzuki (1988, 2003).

**Figure 6.**
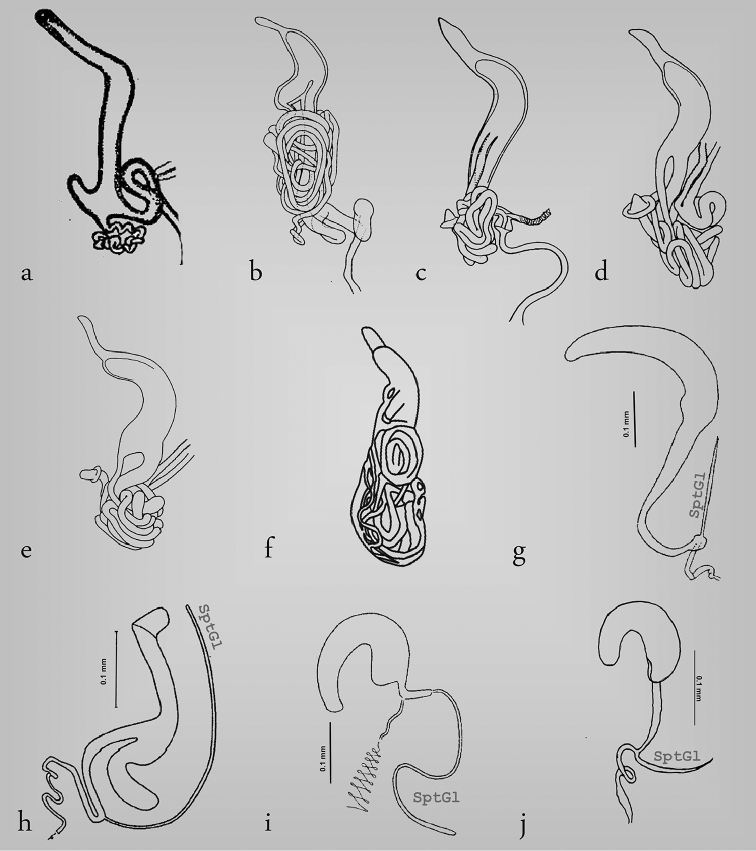
Structure of the spermatheca in Megalopodinae (**a**), Zeugophorinae (**d–f**) and Palophaginae (**g–j**) . **a** *Megalopus* sp. 2 **b** *Zeugophora toroja* **c** Z. *javana* **d** *Z. vitinea* **e** *Z. williamsi* **f** *Zeugophorella riedeli* **g** *Palophagus bunyae* **h** *P. australiensis* **i** *Cucujopsis setifer* **j** *Palophagoides vargasorum*.

**Figure 7.**
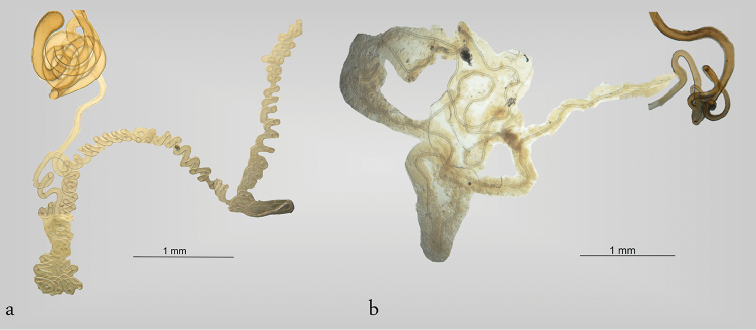
Spermathecal duct and gland in Megalopodidae (Megalopodinae). **a** spermathecal duct of *Homalopterus tristis* **b** spermathecal gland of Agathomerus (Eugathomerus) sellatus.

Our results showed that the three subfamilies of Megalopodidae can be effectively differentiated by their particular spermathecal anatomy (Table 2). We did not find intraspecific variation in the spermatheca. All subfamilies exhibit a spermathecal capsule (SptC), a spermathecal gland (SptGl) and a spermathecal duct (SptD); variations of these structures provide the diagnostic characters for these subfamilies (Fig. 1–6, Table 2). The SptD diameter and length are variable, and the length is always longer than the SptC (Figs 2i, 3a, b, i, 5b, 7a). The SptGl is wide and also longer than SptC (Figs 5a–d, 7b), except in Palophaginae where it is either shorter or the same size as the SptC (Figs 6g–j, Table 2). The SptC has wide walls and it is well sclerotized as in other coleopteran families (Figs 1–4). The shape of the SptC varies among the species of Megalopodidae (Figs 1–6).

**Table 2.** Differences between the subfamilies of Megalopodidae.

	SptC morphology	SptCp	SptGl	SptD	Hold the SptM
**Megalopodidae**	complex	boomerang-shaped	not branched and longer	very long	apex and the stem
**Zeugophorinae**	complex	crane’s neck-shaped	branched and longer	very long	apex and the terminal portion
**Palophaginae**	simple	C-shaped	not branched and short	short	–

In this study the SptC was divided in two portions, the proximal part of spermathecal capsule (SptCp) and the distal spermathecal part (SptCd) (Fig. 1a), following the homologies proposed by Suzuki (1988). The SptCp has a particular shape in each subfamily. Megalopodinae has a boomerang–shaped SptCp (Figs 1–3, 5a–c, 6a); in Zeugophorinae it is crane’s neck–shaped (Figs 4, 5d, 6b–f); and in Palophaginae it is C–shaped (Fig. 6g–j, Table 2). In some species, the SptCp exhibits a prolongation of the apical wall called the velum (Figs 1, 2, 3a, b, 4a-c, 5a, b, 6a-f, h); it is less sclerotized than the rest of the SptC wall. The first part of the SptCd (=stem) (Fig. 1) is variable in length and sometimes is elongate (Figs 1–6). The SptCd has duct–shaped, the terminal portion in its last portion is globose (Figs 1, 2b, f–i). The SptCd is bifurcate (Figs 1, 2, 3a, i, 4a–b, 5a, d), ending the stem; this bifurcation could be a common duct (CoDu) between the SptGl and the SptD (Figs 2g, 3c, 4a, 6h).

In Megalopodinae, the differences among genera are especially evident in the shapes of the SptCp and SptCd. The genera *Agathomerus*, *Homalopterus* Perty, 1832, *Mastostethus*, and *Megalopus* (Figs 1, 2, 3a, b, 5a, 6a) have similar spermathecae; thus, they are proposed in this study as the “American group.” This group has a boomerang–shaped SptCp, with a velum. The SptCd is elongate, wide, and variable in length. The stem in the SptCd is long, and the apex of the SptC holds the spermathecal muscle (SptM) (Figs 1b, 5a). Some species have a stem with ornaments (Figs 2f, g, i). The terminal portion of the SptC in *A. flavomaculatus* (Klug, 1824) and *A. signatus* (Klug, 1824) is coiled and notably long (Figs 2d and 2e respectively); in *Megalopus inscriptus* Klug, 1824 (Fig. 3a) and *Megalopus* sp. 1 (Fig. 3b) it is shorter. The SptD can be wider and short as in *Megalopus* (Figs 3a, b), narrow and long as in *Mastostethus nigrocinctus* (Chevrolat, 1832) (Fig. 2g), or coiled as in *Homalopterus tristis* Perty, 1832 (Fig. 7a).

The apex of the SptCp in *Temnaspis* Lacordaire, 1845 (Figs 3c, d) does not have velum, and the internal part is abruptly narrowed towards the apex. The SptCd has a long stem that can have either two or three ramified ducts, as in *T. speciosus* Baly, 1859 and *T. septemmaculata* (Hope, 1831). These ducts are bifurcate and are connected between each other, forming a complex mass of ducts covering the stem. The CoDu is long and somewhat coiled, and it originates in the terminal portion of the SptC. Finally, the SptD is very variable in length and coils.

The genus *Poecilomorpha* Hope, 1840 has coarse walls in the SptCp, the apex is emarginate and without a velum (Fig. 3f, h), and its internal part is acuminate (Fig. 3e, g). The SptCd in *P. cyanipennis* (Kraatz, 1879) is divided in three branches connected between the SptCd and SptCp (Fig. 3e). The main connection is the stem, and the other two branches attach laterally and are interconnected with the stem; these branches lack ramifications. All the lateral branches of the SptCd are coiled and form a mass. The CoDu has a diameter greater than that of the lateral ducts, and it is attached in the basal portion of SptCd. The SptCd in *P. atripes* Lacordaire, 1845 is globose and short (Fig. 3g), and the CoDu is thicker. The SptC in *Sphondylia* Weise, 1902 is different because of the tetrahedral form of the SptCp. The stem is short and is joined laterally to the terminal portion. The stem and the terminal portion are connected by the CoDu (Fig. 3i) that is globose, thick, and short. Finally, there is no connection with the SptCd.

The structure of the spermatheca in Zeugophorinae is notably different from Megalopodinae. The ventral wall of SptCp is narrow in *Zeugophora californica* Crothc (Fig. 4a), 1874 and *Zeugophora varians* Crothc, 1873 (Fig. 4b). The SptCd is elongate and twisted towards the apex, the stem is short, and the terminal portion in its last portion is fusiform (Fig. 4a, b). The apex and the terminal portion hold the SptM (Fig. 4b). The SptCd in *Zeugophora indica* Jacoby, 1903 (Fig. 4c), *Z. annulata* (Fig. 5d), and *Z. javana* (Fig. 6c) is an elongate and complex structure that is branched into three ramifications coiled in a subspherical mass (Suzuki 1988, Reid 1992). The terminal portion in its last portion presents two parallel structures that hold the SptM (Figs 4c, 5d). The SptGl is branched (Fig. 5d). The SptCd in *Z. toroja* (Fig. 6b), *Z. vitinea* (Fig. 6d), and *Z. williamsi* (Fig. 6e) is somewhat elongate, and it forms two terminal branches and do not form any type of mass. The last portion of SptCd is mound–shaped. Sekerka and Vives (2013) mentioned that *Z. riedeli* (Fig. 6f) has a characteristic velum and a long well coiled duct that is connected many times with the vasculum (= SptCp).

The subfamily Palophaginae (Figs 6g–j) has a simple spermatheca. The SptGl is short and narrow, and the SptCp is variable among the species. *Palophagus bunyae* Kuschel, 1990 (Fig. 6g), *P. australiensis* Kuschel, 1990 (Fig. 6h), and *Palophagoides vargasorum* Kuschel, 1996 (Fig. 6j) have an elongate SptCd. The SptGl and SptD are connected in the terminal portion. *Cucujopsis setifer* Crowson, 1946 (Fig. 6i) has the SptD reduced, and it is connected laterally with the SptCd. *Palophagus bunyae* (Fig. 6g) and *C. setifer* (Fig. 6i) have the SptD very long and coiled (Kuschel and May 1990, 1996).

## Discussion

The structure of the spermatheca in Megalopodidae (Palophaginae + Zeugophorinae + Megalopodinae) is complex, and it is associated with a high diversity in forms. This variability affords characters with great taxonomic and phylogenetic value at various taxonomic levels. The structure of the spermatheca has been used to delimited species, that is the case of the genus *Mastostethus* (Rodríguez-Mirón and Zaragoza-Caballero 2017).

The spermatheca in Megalopodidae consists of a SptC, SptD, and SptGl, which is the arrangement that is the commonest in Coleoptera, including Chrysomeloidea, except in *Vesperus luridus* (Rossi, 1794) (Vesperidae), which does not have an SptD or an SptGl (De Marzo 2008). The SptC of Coleoptera is usually well sclerotized, as in Megalopodidae (Figs 2–4), and this condition that helps with sperm storage (Suzuki 1988, Candan et al. 2010). However, the families Orsodacnidae and Vesperidae have a membranous SptC (Suzuki 1988, Saito 1993).

The SptC has a particular structure in the three subfamilies of Megalopodidae. The morphology of the SptCp and SptCd in Zeugophorinae and Megalopodinae is complex (Suzuki 1988, 2003), similar to that of Disteniidae where the SptC has a complex arrangement in the SptCd, the stem being globose, the SptCp being C–shaped, and the SptC being “?–shaped” (Lin and Murzin 2012, Bi and Lin 2013). In Chrysomelidae, Orsodacnidae and Cerambycidae the SptC is simple, C–shaped or hook–shaped, and the SptCd is wide (Suzuki 1988, Hernández 1993, Hernández and Ortuño 1992, Mergen 2004, Chamorro-Lacayo et al. 2006, Yus-Ramos 2008, Gui-Yi and Li 2012).

The C–shaped SptC is present in Palophaginae (Fig. 6i, j), the sister group of the remaining two subfamilies of Megalopodidae (Reid 1995, Marvaldi et al. 2009). Lamiinae (Cerambycidae) has a narrow SptCd and a wide SptCp (Hernández and Ortuño 1992, Hernández 2000, Lin et al. 2009). The SptCp in Vesperidae is like an elongate sack, and this character is considered a plesiomorphic state (Saito 1993, De Marzo 2008). Considering the last idea, the C–shaped SptC in Megalopodidae (Fig. 6i, j) could be considered as a plesiomorphic state present in a common ancestor of Orsodacnidae, Cerambycidae, Chrysomelidae, and Megalopodidae. Moreover, the complex arrangement of the SptC in Megalopodidae (Figs 2–5, 6a–f) could be considered as an apomorphic state. These changes, from simple to complex structure in the SptC, have been mentioned as an evolutionary change in Cerambycidae (Saito 1993) and Criocerinae (Matsumura et al. 2014).

The shape and length of the SptGl and SptD are not taxonomically or phylogenetically diagnostic among families. These structures should be considered as homoplastic, in view of the heterogeneity in Cerambycidae and Chrysomelidae (see Suzuki 1988, Saito 1993). Even so, the SptGl of Megalopodidae transitions from simple to complex. Palophaginae has a short SptGl (Fig. 6g, j) (Kuschel and May 1990, 1996), in contrast with Zeugophorinae and Megalopodinae (Figs 5a–d, 7b), where it is longer and thicker in comparison to the SptC. The SptGl in Zeugophorinae is branched (Fig. 5d) (Suzuki 1988, 2003).

The SptD in Megalopodidae is characterized by being longer than the SptC (Figs 2i; 3a, b; 5b; 7a). The SptD length has a close relationship with the flagellum length in males. That is the case of *Megalopus armatus* Lacordaire, 1845, where the flagellum goes until the spermatheca and leaves the spermatophore (Flowers and Eberhard 2006). This relationship has been found in some species of leaf beetles (Chrysomelidae), such as in *Chelymorpha alternans* Boheman, 1884 (Cassidinae) (Rodriguez et al. 2004) and in some species of Lema (subgenus Lema) Fabricius, 1798 (Criocerinae) where it is considered as a plesiomorphic state (Matsumura and Suzuki 2008). Also, a relationship between the SptD and the flagellum has been found in Staphylinidae (Gack and Peschke 1994).

The correlation of the lengths of the reproductive organs in Megalopodinae is characteristic of the genus *Megalopus*. However, in the genera *Homalopterus*, *Temnaspis*, and *Agathomerus*, this correlation is obscured because the SptD is very long and coiled (Figs 3c, d, 7a). Moreover, *A. flavomaculatus* (Fig. 2d) and *A. signatus* (Fig. 2e) have a very long SptCd, in contrast to *Megalopus*, where the SptD is shorter and not coiled, and the SptCd is short (Figs 3a, b, 6a). The length of the flagellum has been pointed out as the main factor for fitness, where the selective pressure favors a longer flagellum as a result of sexual selection (e.g. Rodriguez et al. 2004, Matsumura and Suzuki 2008).

The SptM has an important function in reproduction. The SptC in Coleoptera is adapted in many ways to give two places of insertion of the muscle fibers, which form the SptM (De Marzo 2008). The surface of the SptC in Megalopodidae has two forms for connecting the muscle fibers. The first one is present in Megalopodinae, where the fibers connect the apex of the SptC with the stem (Figs 1b, 5a). The second way is where the apex is connected with the terminal portion; it is present in Zeugophorinae (Figs 4b, 5d). The SptM in Palophaginae has not been described.

Some characters in the spermathecae possibly diagnose genera or groups of genera. For example, the arrangement of the SptC is similar within the American group (*Agathomerus*, *Homalopterus*, *Megalopus*, and *Mastostethus*), but is different from that found in *Poecilomorpha*, *Temnaspis*, and *Sphondylia*, because of the presence of a velum in the American group. *Sphondylia* differs from the rest of the genera of Megalopodinae, due to the tetrahedral arrangement of the SptCp (Fig. 3i).

The walls thickness of SptCp have differences among Megalopodinae. The apical portion in *Poecilomorpha* and *Temnaspis* is acuminate (Fig. 3c–e, g). These walls are gradually reduced in the American group (Figs 1, 2, 3a, b).

Within the subfamily Zeugophorinae, there are differences in the SptC. The genus *Zeugophorella* Sekerka, 2013 (Fig. 6f) has multiple connections between the SptCd and the SptCp. Such connections are not present in *Zeugophora* Kunze, 1818. Between the Nearctic species and the Asian species that were sampled in this study, there are differences in the arrangement of the SptC. The North American species (*Z. californica* and *Z. varians*) have a curved and elongate SptCp (Fig. 4a, b). Among the Old World species, *Z. indica*, *Z. annulata*, and *Z. javana* have an SptCd with a complex mass of ducts forming three branches (Figs 4c, 5d, 6c) (Suzuki 1988, Reid 1992). In addition, the structure that holds the SptM is different. In the Nearctic species, it is fusiform (Fig. 4a, b); in *Z. indica* and *Z. annulata* (Figs 4c, 5d), this structure is like two parallel bars, and in *Z. javana* (Fig. 6c), *Z. vitinea* (Fig. 6d), and *Z. williamsi* (Fig. 6e), it is mound–shaped. *Zeugophora annulata* has been treated as part of the subgenus Pedrillia Westwood, 1864, but this subgenus was just synonymized with Zeugophora (Sekerka and Vives 2013). This taxonomic change was made because of the lack of diagnostic characters that validate the subgenus Pedrillia. The spermatheca provides characters to diagnose genera and subgenera. Particularly useful is the SptCd of the SptC, which is different between the North American species and the Asiatic species of *Zeugophora*.

## Conclusions

The present study compares the spermathecae of Megalopodidae, and it considers species from all three subfamilies (Megalopodinae, Zeugophorinae and Palophaginae). It describes for the first time this structure for 20 taxa.

We conclude that the SptCp variations are informative and useful in diagnosing these three subfamilies. In addition, the variations observed in the distal portion of the SPtCd are diagnostic of several genera, and, in some cases, groups of genera, such as the American group.

Finally, we believe that the spermatheca has a high taxonomic value for diagnosing taxa at various ranks within Megalopodidae. However, further testing of this hypothesis, to be provided by phylogenetic analyses, will establish the phylogenetic signal and corroborate the homology hypothesis of this character complex.
